# Influence of Cleft Lip and Palate on Oral Health-Related Quality of Life in Northern Italy: Exploring Both the Children’s and Caregivers’ Perspectives

**DOI:** 10.3390/children9121986

**Published:** 2022-12-17

**Authors:** Patrizia Defabianis, Rossella Ninivaggi, Federica Romano

**Affiliations:** Department of Surgical Sciences, C.I.R. Dental School, University of Turin, 10126 Turin, Italy

**Keywords:** oral health-related quality of life, rare disease, orofacial cleft, children, caregivers, parents, Child Oral Health Impact Profile

## Abstract

The aim of this cross-sectional study was to examine whether parents/caregivers’ perceptions of oral health-related quality of life (OHRQoL) differ from that of their cleft children, exploring the impact of demographic variables and cleft type on their agreement. Fifty-three primary and secondary schoolchildren, with non-syndromic orofacial cleft, and their parents answered the Child Oral Health Impact Profile (COHIP) questionnaire. Concordance between caregivers’ and children’s reports was low to moderate. Parents/caregivers had worse perceptions of OHRQoL compared to that of their children, peer interaction and functional well-being domains being statistically significantly different (*p* = 0.033 and *p* = 0.005, respectively). Cleft type, gender and parents’ country of origin seemed to be potential contributing factors of disagreement. Parents overestimated the impact of unilateral (*p* = 0.047) and bilateral cleft lip and palate (*p* = 0.021) on OHRQoL, and they rated more poorly than their male children did. Italian parents were more concerned about the functional well-being of their children (*p* = 0.014), and foreign parents about peer interaction (*p* = 0.010) and school environment (*p* = 0.012) dimensions. These findings suggest that parental assessment of OHRQoL cannot replace that of school-aged children, but they are complementary as they cover different, but equally relevant perspectives.

## 1. Introduction

Cleft lip, with or without palate, (CL ± P) is one of the most common congenital craniofacial anomalies resulting from failure of embryonic fusion processes during embryogenesis due either to genetic or environmental factors [1]. According to a recent systematic review, in 2017 the worldwide estimated incidence of CL ± P was 195,500 [2]; about 71% of cases were isolated and 29% were syndromic and associated with other genetic abnormalities [3].

CL ± P represents a relevant public health issue because of the complex surgical and rehabilitative treatment extending from infancy to adulthood, the long-term consequences on oral health and the psychosocial implications for daily life [4]. The abnormal craniofacial growth and the presence of dental anomalies can result in oro-facial malocclusions, such as open bite, crossbite, crowding and skeletal Class III, which may impact on esthetics and functions, such as speech and swallowing [5,6,7,8]. Furthermore, in recent years it has become more and more evident that the CL ± P condition may severely impact on oral health-related quality of life (OHRQoL) [9,10]: children and adolescents with CL ± P report worse social, functional and psychological well-being in everyday life than their non-cleft peers do [11,12,13,14].

Considering OHRQoL as an important auxiliary tool to evaluate health in addition to traditional clinical aspects, it is fundamental to take into account the reliability of children’s opinions regarding their own well-being. It has been suggested that both children and parents should report ratings of OHRQoL using appropriate questionnaires [15]. The Child Oral Health Impact Profile (COHIP), developed to evaluate OHRQoL in the dimensions of oral health, functional well-being, school environment and peer interaction in 8–15-year-old children [16], also proved to be valid and reliable in CL ± P subjects [17]. In addition, a corresponding COHIP questionnaire was developed for parents/caregivers. Even though some surveys report no differences in OHRQoL between the scores of parents and children [12,18,19], others underline the existence of discrepancies in the overall COHIP and in the subscales [14,15]. Under/over-estimation of the child’s aesthetics, symptoms, biases and expectations regarding the treatment results may affect the perception of caregivers; on the other hand, children’s perspectives may be altered by the dominance of short-time memory, the influence of recent incidents and language or reading problems [15].

So far, in Italy, an analysis of the impact of CL ± P on the quality of life of patients and parents/caregivers is still missing, and this evaluation could be useful in allowing healthcare providers to have a full picture of the healthcare needs of these patients. Therefore, the aim of this cross-sectional study was to investigate whether parents/caregivers can accurately rate the OHRQoL of their school-aged children with CL ± P using the COHIP questionnaire. The second aim was to get more information on the impact that demographic characteristics and cleft type have on the agreement between children and parents: for this purpose, we hypothesized that both COHIP reports were interchangeable.

## 2. Materials and Methods

### 2.1. Study Design and Participants

This mono-centre cross-sectional study was carried out at the Section of Paediatric Dentistry, C.I.R. Dental School, University of Turin (Italy) from December 2019 to March 2022 in according with the Helsinki Declaration. Ethical consent for the study was obtained by the Institutional Ethical Committee of the “AOU Città della Salute e della Scienza” of Turin (n. 0038526).

The study population included consecutive non-syndromic children either with unilateral or bilateral cleft lip (CL), or unilateral (UCLP) or bilateral cleft lip and palate (BCLP), or isolated cleft palate (CP) and their parents/caregivers. Only children aged 8– 14 years were included; additional medical diagnosis, intellectual disability and craniofacial malformation were excluding criteria. All children and parents/caregivers needed to have complete command of the Italian language to be able to answer the questionnaire appropriately. They were informed about the aim of the study and of the whole procedure, as well as about pseudonymized data collection to protect their privacy throughout the study. Written consent was received from parents/caregivers of all participants prior to enrolment.

### 2.2. Data Collection and COHIP Questionnaire

Data on age, gender, ethnicity, type and side of the cleft, presence of any concomitant systemic pathology and parents’ country of origin were recorded.

All patients and parents/caregivers were asked to answer the COHIP questionnaire on the same day, during a routine dental appointment; all completed the Italian version of the questionnaire independently, in separate rooms [20]. The COHIP consisted of two parallel questionnaires, one for children and one for parents, which were identical, but based on different perspectives. Both inquiries were organized into five subscales (oral symptoms, functional well-being, social/emotional well-being, school environment, and peer interaction) for 34 items referring to positive or negative children’s experiences in the last 3 months.

Every item was rated on a five-point Likert scale (from 0 = never to 4 = almost all the time), with the additional response option ‘I don’t know’ that was scored as missing. In order to make the results easier to interpret, the negatively formulated items were reverse coded before analysis, so that the higher the COHIP values, the better the OHRQoL. The subscale scores were added together to give the overall OHRQoL, ranging from 0 (the worst OHRQoL) to 136 (the best OHRQoL). Questionnaires less than 75% completed were excluded from the evaluation.

### 2.3. Statistical Analysis

Quantitative data were summarized as mean and standard deviation or median and range (minimum–maximum); categorical data were presented as absolute and relative frequencies. The reliability of the answers was examined using Cronbach’s alpha. Comparisons between overall and subscale COHIP scores of affected children and those of parents/caregivers were performed using the Wilcoxon test. Intraclass correlation coefficients (ICCs) between subscales of children and parents were also computed.

An additional analysis was carried out to test the influence of demographic characteristics and cleft type on the OHRQoL perception of parents and cleft children. Children were divided into 3 subgroups according to their age: 8–10, 11–12 and 13–14 years. Parents were categorized as either born in Italy or born in a foreign country. A stratified analysis was carried out to test the effect of children’s gender (boy, girl) and age (8–10 years, 11–12 years and 13–14 years), parent’s country (Italian and foreign) and cleft type (CL, CP, UCLP, BCLP) on the similarity between parents and children in the total and subscale COHIP scores using the Wilcoxon test. All data were analysed using SPSS software (26.0; IBM Inc.). The level of significance was set at 0.05 (*p* < 0.05).

## 3. Results

Fifty-three primary and secondary schoolchildren with CL ± P and their parents/caregivers completed the COHIP questionnaires. All the children received multidisciplinary cleft care at the Regina Margherita Hospital, a specialist centre for the treatment of orofacial anomalies Northern Italy.

The demographic characteristics of the study population are shown in Table 1. More than half of the patients were male (54.7%) and most of the participants had CLP (17.0% bilateral and 56.6% unilateral). The mean age was 11.1 ± 2.0 years (range 8–14 years). The caregiver questionnaires were most often filled in by the parents (79.2% mothers, 13.2% fathers), with only 4 of them (7.5%) being completed by other caregivers.

The internal consistency of the COHIP questionnaire was good for both parents and children with Cronbach’s alpha coefficients of 0.859 and 0.855, respectively.

As reported in Table 2, the mean COHIP score for children was 98.5 and the median was 102; the mean score for parents was 92.0 and the median was 94.0. All subscales of the parents’ COHIP but not those of the children’s questionnaire had the floor scores (maximum effect on OHRQoL), whereas the ceiling scores (minimum effect on OHRQoL) were found in all the subscales, except for oral symptoms in the cleft group and social emotional and school environment dimensions in the parental group. Parents scored lower than children in most of the subscales, but differences reached statistical significance in the overall COHIP (*p* = 0.024) and in the subscales functional well-being (*p* = 0.005) and peer interaction (*p* = 0.033).

The ICCs suggested low to moderate agreement between child and parent reports (Table 3). The lowest ICCs were found related to the oral health and functional well-being dimensions, while the higher ICCs were related to the social emotional domain.

A stratified analysis was performed to explore the impact of a child’s gender (Table 4), age (Table 5), parent’s country of origin (Table 6) and cleft type (Table 7) on the concordance between children and parents in the COHIP scores. The parents of boys tended to score significantly lower than their male children in the domains of functional well-being (*p* = 0.004), social-emotional well-being (*p* = 0.023) and peer interaction (*p* = 0.018), whereas the parents of girls had a perception of OHRQoL more similar to that of their daughters (Table 4).

The parents’ perceptions of the peer interaction and functional well-being subscales were statistically significantly different from those of their children in the 8–10 and 13–14-year age groups, respectively (both *p* = 0.001, Table 5). Conversely, no differences were observed between the parents’ and children’s ratings in the 11–12-year age group.

Italian parents seemed to be more worried about the functional well-being of their children (*p* = 0.014), whereas foreign parents seemed to be more worried about the psychological dimensions of school environment (*p* = 0.012) and peer interaction (*p* = 0.010) (Table 6).

Table 7 shows the comparisons between the COHIP scores reported by parents and children according to the type of cleft. Owing to the small sample size in some cleft categories, only explorative data were presented. Cleft type was found to have an impact on parent and child ratings only for BCLP and UCLP.

## 4. Discussion

The primary aim of the present cross-sectional study was to assess child–caregiver concordance regarding the impact of orofacial clefts on children’s OHRQoL using the COHIP tool. The study included fifty-three non-syndromic CL ± P children, aged 8–14 years, who had received multidisciplinary intervention care and attended a university-based dental clinic in Northern Italy for routine dental consultation.

The present findings indicated low to moderate concordance between patients’ and caregivers’ reports with a tendency for parents to rate the OHRQoL of their children lower than children did in most of the examined domains. The lowest ICCs were found related to the oral health and functional well-being dimensions. Thus, the null hypothesis was rejected, suggesting that parents are not a suitable proxy for evaluating their children OHRQoL. Among the OHRQoL measurement instruments [21], we used the COHIP questionnaire because it was specifically designed for this age category and it combines a self-report from the child and a proxy one from the caregiver [16]. Furthermore, it was found to be an appropriate and reliable tool for children with craniofacial anomalies [17,22]. Consistently, we obtained a good internal consistency for both children and caregivers [16].

A previous study using the COHIP tool found a similar tendency towards worse scores among parents of American children aged 8–15 years [15]. Similarly, Leopoldo-Rodado et al. in Spain [14] and Kramer et al. in Germany [23] demonstrated lower scores for parents compared to 4–7-year-old or 8–12-year-old children, respectively, using the KINDL questionnaire, but they did not report any data on the level of agreement. In contrast, Ward et al. [12] found no significant differences in the overall COHIP, or in any of the subscales, between parents and children. Other studies confirmed high rates of similarity and comparable COHIP scores between children and parents [18,24]. Even though the rate of concordance was high, Bos & Prahl [11] and Nolte et al. [25] found significant differences in the Dutch population in the mean scores of functional well-being, emotional well-being, oral symptoms and school domains. This suggests that data from between-group comparisons of average total/subscales scores are complementary to the ICC analysis, which measures the intra-individual variations between each parent-child dyad.

We observed statistically significant differences between children and parents in the peer interaction and functional well-being subscales, with parents rating the difficulties as more severe than children did. According to other reports [15,25] it appears that parents ascribe more influence to the functional inconvenience of having a cleft on the quality of life of their children. Notably, this dimension includes questions on word pronunciation and intelligibility. Speech difficulties are often complained about by cleft patients and are due to the structural anomalies of the soft palate affecting the emphasis and articulation of certain sounds [26,27]. In cases of velopharyngeal insufficiency the affected children can also suffer from hypernasal resonance [27].

In contrast to previous reports showing that parents underestimate the impact of the oral conditions on children’s emotional quality of life, in our study they are highly concerned about the difficulties experienced by their children in their social integration [13,28]. Jardine et al. [29] emphasized that subjective domains are the most challenging for proxy versus children agreement. Children with CL ± P are more likely to develop psychosocial problems including anxiety, depression, learning and behavioural problems at school, less acceptance by peers, less ability to maintain relationships due to their facial appearance and speech difficulties [30,31].

As a second aim of the study, a more in-depth analysis was undertaken to assess the possible effects of sociodemographic variables and cleft phenotype on parent versus child COHIP scores. Interestingly, Italian parents were more worried about the functional limitations of having CL ± P, whereas foreign parents were more worried about the social implications, both in the school environment and in peer relationships. This might be due to cultural differences, which might have resulted in different attitudes and expectations [18,32].

Gender seemed to be a potential contributing factor in parent-child disagreement. Parents of boys scored worse than their children in the overall COHIP, functional well-being, peer interaction and social environment domains, whereas parents of girls gave similar responses to those of their daughters. These findings may be explained by the different sex-related social demands and attitudes at school ages. Kramer et al. reported that most of the OHRQoL limitations in boys with orofacial clefts represented difficulties in social interactions with peers or caregivers [23]. Based on parents’ reports, Collett et al. [33] found that males with cleft experienced more behavioural problems compared to their peers, whereas females did not. Girls tend to reveal their problems more to other people and to provide their parents with more detailed information [34]. In contrast, boys are less willing to ask for help, they prefer to cope with their problems on their own and are more often victims of bullying and social exclusion, so are more likely to experience higher distress [35]. Thus, CL ± P boys seem to have a more serious impact on family functioning than girls [23]; furthermore, a child’s chronic illness results in increasing stress and decreased communication levels in the family over time [36].

It is worth noting that child’s age had a limited effect on the similarity of the responses. The only two domains in which parents rated worse than their children were the peer interaction and functional well-being domains, the scores of which were statistically significantly different from those of children aged 8–10 years and 13–14 years, respectively. It could be argued that, with increasing age children are progressively more and more able to communicate their emotional issues to the family, which unfortunately is perceived by parents only to a limited extent [37]. Furthermore, it is worth noting that, in the current study, most of the people in the parental group were mothers, which did not allow for statistical comparisons between the proxy reports of mothers and fathers. Previous studies have reported that the fathers and mothers of affected children differed in psychological well-being and adjustment, with fathers showing higher self-esteem and less concern about negative judgement from others [38].

Finally, we found an impact of cleft phenotype on OHRQoL perception only for BCLP and UCLP. This finding corroborates previous reports showing lower OHRQoL scores among parents having children with CL or CLP (a visible facial difference) compared to those of children with isolated CP (no facial difference) [39,40]. In contrast, Nidey et al. did not find any difference in parental psychosocial characteristics due to cleft type [38]. Broder et al. found a negative association between the number of previous surgeries and child/caregiver rated COHIP scores [40]. It can be argued that children with more severe defects, such as BCLP and UCLP, had undergone a higher number of cleft-related surgical interventions.

The current study acknowledges some limitations. Owing to the cross-sectional design, the data presented are able to identify the impact of sociodemographic and clinical variables on parent/child OHRQoL agreement at only one point in time. Issues related to sociocultural differences in terms of educational level, ethnicity and socioeconomic status might be relevant components that warrant further study in this population. Another limitation is the small sample size, due to the single-center recruitment and the low incidence of CL ± P [2]. Consequently, even the comparison among different types of clefts has to be drawn with some caution due to the small number of children included in some cleft groups.

Finally, the present data refer to a specialized cleft center in Northern Italy, reducing the extensibility of the results to other populations. According to Collett et al. [33], it should be also taken into account that differences in psychosocial functioning among cleft children and their parents/caregivers may be more evident in a clinical-based versus a population-based sample.

## 5. Conclusions

Overall, parents and their 8–14-year-old children rated the impact of cleft condition on quality of life differently using the COHIP questionnaire. Caregivers reported worse scores in the peer interaction and functional well-being domains than their children did. Gender, type of cleft and parents’ country of origin were the most relevant contributing factors of disagreement between parents and children. These findings suggest that parents cannot replace children in assessing their OHRQoL, but can provide only complementary information. Both children with non-syndromic CL ± P and their parents/caregivers need the targeted support of health professionals and family members in order to improve their well-being. Future research, including a larger sample size and possibly multi-centre studies, would be useful to investigate the long-term functional and psychological effects of CL ± P on OHRQoL in Italy.

## Figures and Tables

**Table 1:** General characteristics of the sample.

Variables	
Age, mean ± SD (years)	11.1 ± 2.0
Age category, *n* (%)	
<8–10 years	20 (37.7)
11–12 years	20 (37.7)
13–14 years	13 (24.6)
Gender, male/female	29/24
Ethnicity, *n* (%)	
Caucasian	43 (81.1)
Chinese	4 (7.6)
Hispanic	6 (11.3)
Type of cleft, *n* (%)	
CL	6 (11.3)
UCLP	30 (56.6)
BCLP	9 (17.0)
CP	8 (15.1)
Parents/Caregivers, *n* (%)	
Mother	42 (79.2)
Father	7 (13.2)
Other	4 (7.5)
Parents’ country *n* (%)	
Italy	43 (81.1)
Foreign	10 (18.9)

BCLP, Bilateral cleft lip and palate; CL, cleft lip; CP, isolated cleft palate; UCLP, unilateral cleft lip and palate on the left or right side.

**Table 2:** Comparison of COHIP overall and subscale scores between cleft children and parents.

COHIP (Maximum Possible Score)	Group				*p* Value
	Cleft Children (*N* = 53)		Parents/Caregivers (*N* = 53)		
	Mean ± SD	Median (range)	Mean ± SD	Median (range)	
Overall COHIP (136)	98.5 ± 16.6	102.0 (62/131)	92.0 ± 19.5	94.0 (0/127)	0.024
Oral symptoms (40)	27.2 ± 4.9	27.0 (12/38)	26.3 ± 5.6	27.0 (0/37)	0.484
Functional well-being (24)	17.9 ± 3.3	18.0 (9/24)	15.6 ± 5.1	16.0 (0/23)	0.005
Social emotional (32)	24.9 ± 7.5	28.0 (5/32)	23.4 ± 7.4	25.0 (0/32)	0.153
School environment (16)	12.4 ± 2.6	13.0 (5/16)	12.3 ± 2.8	13.0 (0/16)	0.733
Peer interaction (24)	16.2 ± 5.5	16.0 (5/24)	14.3 ± 4.2	14.0 (0/22)	0.033

**Table 3:** Intraclass Correlation Coefficients (ICCs) for COHIP overall and subscale scores between cleft children and parents.

Overall and Subscale	ICC	95% CI	*p* Value
Overall	0.517	(0.163 to 0.721)	0.005
Subscales			
Oral symptoms	0.313	(−0.191 to 0.603)	0.090
Functional well-being	0.383	(−0.069 to 0.644)	0.042
Social emotional	0.671	(0.430 to 0.801)	<0.001
School environment	0.580	(0.272 to 0.757)	0.001
Peer interaction	0.582	(0.276 to 0.759)	0.001

CI, interval confidence.

**Table 4:** Effect of children’s gender on the agreement in COHIP overall and subscale scores between parents and children with cleft [mean ± SD, median, (range)].

Gender	Overall COHIP	Oral Symptoms	Functional Well-Being	Social Emotional	School	Peer Interaction
**Female (*N* = 24)**						
Children	95.7 ± 17.3 101.0 (62/117)	26.1 ± 4.1 27.0 (19/35)	17.4 ± 3.2 18.0 (10/22)	24.2 ± 8.6 27.5 (5/32)	12.2 ± 2.7 13.0 (5/16)	15.8 ± 5.9 15.5 (5/24)
Parents/ Caregivers	95.2 ± 16.5 95.0 (61/127)	26.5 ± 5.1 26.5 (14/37)	16.3 ± 4.9 17.0 (5/23)	24.6 ± 7.2 27.0 (11/32)	12.6 ± 2.3 13.0 (9/16)	15.2 ± 3.7 14.5 (10/22)
*p* Value	0.742	0.589	0.430	0.778	0.523	0.660
**Male (*N* = 29)**						
Children	100.9 ± 15.9 105.0 (70/131)	28.1 ± 5.4 29.0 (12/38)	18.3 ± 3.4 19.0 (9/24)	25.4 ± 6.6 28.0 (10/32)	12.6 ± 2.6 12.0 (7/16)	16.4 ± 5.2 16.0 (7/24)
Parents/ Caregivers	89.4 ± 21.6 92.0 (0/119)	26.1 ± 6.1 27.0 (0/34)	15.0 ± 5.2 16.0 (0/23)	22.5 ± 7.5 23.0 (0/32)	12.1 ± 3.2 13.0 (0/16)	13.7 ± 4.5 14.0 (0/21)
*p* Value	0.002	0.185	0.004	0.023	0.312	0.018

**Table 5:** Effect of children’s age on the agreement in COHIP overall and subscale scores between parents and cleft children [mean ± SD, median, (range)].

Age	Overall COHIP	Oral Symptoms	Functional Well-Being	Social Emotional	School	Peer Interaction
**8–10 years (*N* = 20)**						
Children	103.3 ± 12.3 106.0 (71/118)	27.3 ± 3.5 28.0 (22/32)	16.8 ± 3.4 18.0 (9/22)	27.2 ± 5.5 29.0 (11/32)	12.6 ± 2.1 13.0 (7/15)	19.5 ± 3.7 21.0 (11/24)
Parents/ Caregivers	94.38± 13.4 97.0 (70/132)	25.8 ± 4.7 26.0 (14/33)	16.2 ± 3.7 16.5 (10/22)	25.5 ± 5.9 27.0 (12/32)	12.3 ± 2.2 12.0 (8/16)	15.2 ± 3.8 15.0 (8/21)
*p* Value	0.005	0.158	0.657	0.146	0.403	0.001
**11–12 years (*N* = 20)**						
Children	97.4 ± 17.9 97.5 (62/127)	27.0 ± 5.9 27.5 (12/35)	18.8 ± 2.9 18.0 (14/24)	24.7 ± 8.0 26.5 (5/32)	12.2 ± 3.2 12.0 (5/16)	14.9 ± 5.2 15.0 (5/24)
Parents/ Caregivers	99.0 ± 14.6 100.5 (74/127)	27.7 ± 4.2 27.0 (21/37)	19.0 ± 2.7 18.0 (14/23)	24.9 ± 6.2 25.5 (13/32)	12.7 ± 2.5 13.0 (9/16)	14.8 ± 3.4 14.5 (10/20)
*p* Value	0.641	0.669	0.895	0.955	0.557	0.827
**13–14 years (*N* = 13)**						
Children	92.9 ± 19.4 85.0 (65/131)	27.4 ± 5.4 25.0 (19/38)	18.3 ± 3.4 19.0 (10/23)	21.7 ± 8.9 23.0 (5/32)	12.5 ± 2.7 14.0 (7/16)	13.1 ± 5.9 14.0 (5/23)
Parents/ Caregivers	76.9 ± 26.2 88.0 (0/102)	25.1 ± 8.4 27.0 (0/33)	9.5 ± 4.1 10.0 (0/16)	18.2 ± 9.0 21.0 (0/31)	11.9 ± 4.0 13.0 (0/15)	12.3 ± 5.4 12.0 (0/22)
*p* Value	0.060	0.649	0.001	0.212	0.498	0.798

**Table 6:** Effect of parents’ country on the agreement in COHIP overall and subscale scores between parents and children with cleft [mean ± SD, median, (range)].

Country	Overall COHIP	Oral Symptoms	Functional Well-Being	Social Emotional	School	Peer Interaction
**Foreign (*N* = 10)**						
Children	100.5 ± 13.4 101.5 (79/117)	26.3 ± 3.2 26.5 (21/30)	17.5 ± 1.6 17.5 (15/20)	26.1 ± 5.7 28.5 (18/32)	13.4 ± 1.7 14.0 (11/15)	17.2 ± 5.2 16.0 (9/24)
Parents/ Caregivers	85.0 ± 14.7 87.0 (61/103)	24.7 ± 3.9 22.5 (21/32)	14.8 ± 5.6 15.5 (5/23)	22.6 ± 7.2 23.0 (11/32)	11.2 ± 1.3 11.0 (9/13)	11.7 ± 3.0 10.5 (8/17)
*p* Value	0.008	0.171	0.182	0.208	0.012	0.010
**Italian (*N* = 43)**						
Children	98.1 ± 17.4 102.0 (62/131)	27.4 ± 5.2 27.0 (12/38)	18.0 ± 3.6 19.0 (9/24)	24.6 ± 7.9 27.0 (5/32)	12.2 ± 2.8 13.0 (5/16)	15.9 ± 5.6 16.0 (5/24)
Parents/ Caregivers	93.6 ± 20.3 95.0 (0/127)	26.7 ± 6.0 27.0 (0/37)	15.7 ± 5.0 16.0 (0/23)	23.7 ± 7.5 25.0 (0/32)	12.6 ± 3.0 13.0 (0/16)	15.0 ± 4.2 15.0 (0/22)
*p* Value	0.242	0.810	0.014	0.287	0.375	0.329

**Table 7:** Effect of cleft type on the agreement in COHIP overall and subscale scores between parents and children [mean ± SD, median, (range)].

Type of Cleft	Overall COHIP	Oral Symptoms	Functional Well-Being	Social Emotional	School	Peer Interaction
**CL (*N* = 6)**						
Children	96.7 ± 27.1 99.5 (65/131)	28.0 ± 6.5 28.0 (19/38)	18.2 ± 4.3 19.5 (10/22)	22.0 ± 10.9 25.0 (5/32)	12.2 ± 3.9 13.5 (5/16)	16.3 ± 7.5 19.0 (6/23)
Parents/ Caregivers	95.3 ± 15.8 92.5 (75/122)	28.3 ± 2.7 28.0 (25/33)	13.3 ± 5.7 13.5 (7/22)	24.8 ± 7.2 26.5 (13/31)	12.5 ± 3.1 12.5 (9/16)	16.3 ± 5.0 17.0 (10/22)
*p* Value	0.917	0.786	0.058	0.500	0.750	0.916
**UCLP (*N* = 30)**						
Children	98.3 ± 15.3 101.5 (62/127)	27.0 ± 4.9 27.5 (12/35)	18.2 ± 3.1 18.5 (9/24)	24.8 ± 6.8 25.0 (10/32)	11.9 ± 2.7 12.0 (7/16)	16.3 ± 5.6 16.0 (5/24)
Parents/ Caregivers	90.8 ± 22.3 94.5 (0/123)	25.5 ± 6.1 26.0 (0/33)	15.9 ± 5.3 17.0 (0/23)	23.3 ± 8.0 25.0 (0/32)	12.4 ± 3.2 13.0 (0/16)	13.8 ± 4.2 14.0 (0/21)
*p* Value	0.047	0.357	0.036	0.354	0.364	0.029
**BCLP (*N* = 9**)						
Children	98.1 ± 16.5 101.0 (71/118)	26.4 ± 4.1 26.0 (22/34)	16.8 ± 2.8 17.0 (13/21)	24.7 ± 6.6 25.0 (11/32)	13.4 ± 1.9 13.0 (11/16)	16.8. ± 4.8 15.0 (9/24)
Parents/ Caregivers	86.2 ± 15.6 85.0 (70/111)	25.5 ± 6.0 26.0 (14/34)	14.2 ± 3.5 14.0 (8/19)	20.8 ± 7.1 21.0 (12/32)	12.5 ± 1.4 13.0 (11/15)	13.1 ± 3.1 14.0 (8/18)
*p* Value	0.021	0.443	0.172	0.108	0.185	0.043
**CP (*N* = 8**)						
Children	101.4 ± 15.2 103.0 (69/120)	28.0 ± 5.3 28.5 (21/35)	17.6 ± 3.9 17.5 (10/22)	27.4 ± 9.1 30.5 (5/32)	13.5 ± 1.6 13.5 (11/16)	14.9 ± 4.7 15.0 (5/21)
Parents/ Caregivers	100.5 ± 13.3 100.5 (84/127)	28.6 ± 5.0 28.5 (21/37)	17.4 ± 4.9 18.0 (9/23)	26.2 ± 5.0 27.0 (17/32)	11.7 ± 2.0 12.0 (9/15)	16.5 ± 3.8 18.0 (10/21)
*p* Value	0.980	0.399	0.888	0.352	0.057	0.204

BCLP, Bilateral cleft lip and palate; CL, cleft lip; CP, isolated cleft palate; UCLP, unilateral cleft lip and palate on the left or right side.

## Data Availability

The dataset used and analysed during the current study is available from the corresponding author on reasonable request.
